# Case Report: Simultaneous hepatic and renal echinococcosis in a child: a multidisciplinary approach

**DOI:** 10.3389/fped.2025.1623294

**Published:** 2025-07-18

**Authors:** Quanyu Zhou, Yuxiao Xia, Haibo Zou

**Affiliations:** ^1^ Department of Hepatobiliary and Pancreatic Surgery, Chengdu 363 Hospital Affiliated to Southwest Medical University, Chengdu, Sichuan, China; ^2^ Department of Nuclear Medicine, the Second Affiliated Hospital of Chengdu Medical College, Chengdu, Sichuan, China

**Keywords:** pediatric echinococcosis, hepatic echinococcosis, renal echinococcosis, multidisciplinary team, surgical treatment

## Abstract

**Introduction:**

Echinococcosis caused by *Echinococcus granulosus* is a significant public health issue, particularly in pastoral regions. This zoonotic disease is globally distributed and most prevalent in areas with frequent human-livestock interactions. The liver is the most commonly affected organ, followed by the lungs. In children, simultaneous involvement of multiple organs is rare due to immature immune systems and smaller organ sizes. This case highlights the rarity and complexity of simultaneous hepatic and renal echinococcosis in a child.

**Methods:**

A 5-year-old boy from a pastoral region in Tibet, China, was admitted due to a palpable abdominal mass detected one month prior. Imaging studies, including CT, identified multiple echinococcal lesions in the liver and a large cystic mass in the kidney. Serological assays confirmed *Echinococcus granulosus* infection. A multidisciplinary team (MDT) discussion involving pediatricians, anesthesiologists, urologists, and infectious disease specialists developed a tailored surgical plan.

**Results:**

The patient underwent radical multiple pericystectomies of the liver and endocystectomy of the right renal echinococcal cyst. Intraoperatively, multiple firm, translucent masses in the liver and a large cystic mass in the right kidney were found, both confirmed to be infected with *Echinococcus granulosus*. Postoperatively, transient hypernatremia and hepatic dysfunction occurred but were effectively managed. The patient was discharged on postoperative day 7 and showed no recurrence at the 1-month follow-up.

**Discussion:**

This case underscores the complexity and rarity of simultaneous hepatic and renal echinococcosis in children. Early diagnosis through detailed medical history, imaging studies, and serological assays is crucial. The multidisciplinary approach, including tailored surgical strategies and postoperative management, was essential for a favorable outcome. The success of pericystectomy highlights the importance of organ-sparing techniques in pediatric patients. However, potential complications like transient hypernatremia emphasize the need for vigilant postoperative monitoring and supportive care. Future research should focus on improving diagnostic precision, surgical methodologies, and postoperative care in children.

## 1 Introduction

Echinococcosis, a parasitic infection caused by *Echinococcus granulosus sensu lato*, represents a significant public health challenge. This is especially true in pastoral regions where livestock grazing is the primary economic activity (1). This zoonotic disease is globally distributed, with a heightened prevalence in areas characterized by frequent human-livestock interactions. Such areas include the Mediterranean, the Middle East, Australia, New Zealand, South Africa, and South America. Genotyping studies of *Echinococcus granulosus* have demonstrated that the G1 and G3 genotypes are the most prevalent (2). These genotypes exhibit diversity across various regions and host species. The primary hosts for E. granulosus are domestic animals, notably ungulates, while humans act as incidental hosts. Infection in humans typically occurs through the ingestion of parasite eggs present in contaminated food or water. The liver is the organ most commonly affected by echinococcosis, followed by the lungs (3, 4). Other organs, including the kidneys, spleen, bones, and central nervous system, may also be involved. In pediatric cases, the simultaneous involvement of multiple organs is exceedingly rare. This is due to the immature immune systems and smaller organ sizes in children (5, 6). Generally, echinococcosis in children tends to involve a single organ, with the liver being the most frequently affected, due to its role as a filter for circulating parasites. This report documents an uncommon instance of simultaneous hepatic and renal echinococcosis in a 5-year-old patient. It emphasizes the critical importance of early diagnosis, tailored surgical intervention, and diligent postoperative surveillance for complications. It highlights the intricate challenges associated with managing such cases and advocates for further research to improve diagnostic precision, surgical methodologies, and postoperative care in children.

## 2 Case presentation

**Figure 1:**
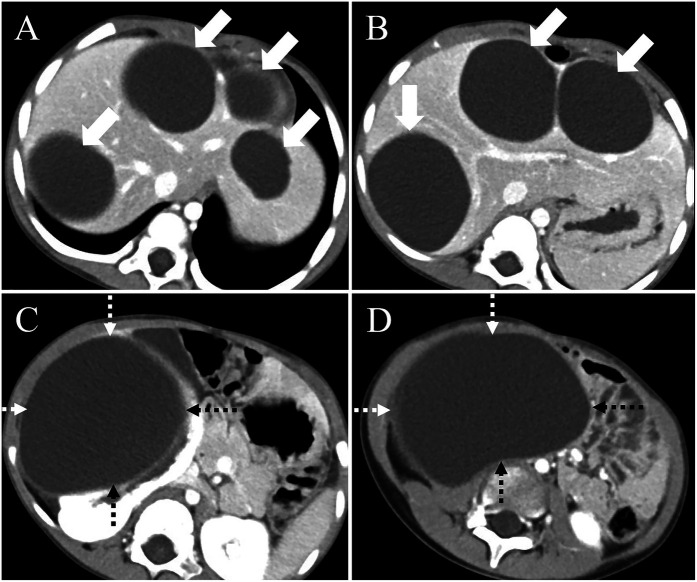
Preoperative computed tomography (CT) findings. **(A)** CT imaging of the hepatic hilum region shows hypodense masses in liver segments 2, 3, 4, and 7 (arrows). **(B)** CT examination of the first hepatic portal region reveals multiple large cystic lesions within the liver (arrows). **(C)** A large cystic lesion is observed in the right kidney (dashed arrow), with the largest cross-sectional area measuring approximately 11.3 × 8.9 cm, and focal discontinuity is noted in the right kidney. **(D)** Pelvic CT scan demonstrates a large cystic lesion originating from the right kidney (dashed arrow).

**Figure 2:**
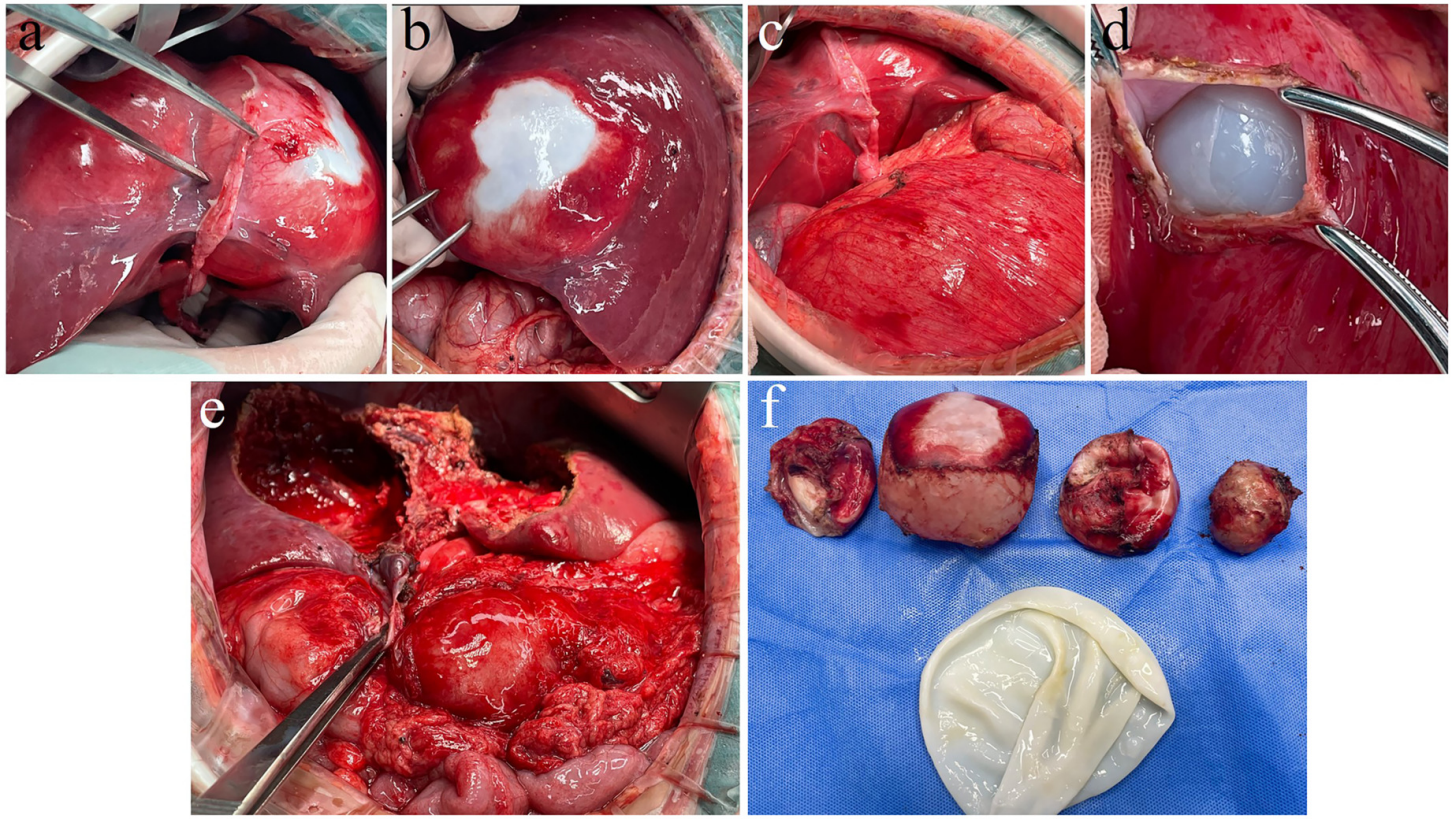
Intraoperative findings of hepatic and renal hydatid cystectomy. **(a)** Hydatid cysts identified in liver segments 2, 3, and 4. **(b)** Following mobilization of the liver ligaments, a large hepatic hydatid cyst in the posterior segment 7 of the right liver lobe is exposed. **(c)** After an upper abdominal reverse “L”-shaped incision, a large renal hydatid cyst is visualized within the abdominal cavity. **(d)** During renal hydatid cystotomy, the endocyst is exposed and the renal hydatid lesion is treated with endocyst enucleation. **(e)** Appearance of the hepatic hydatid lesion following pericystectomy. **(f)** The resected hepatic hydatid lesion specimen.

**Figure 3:**
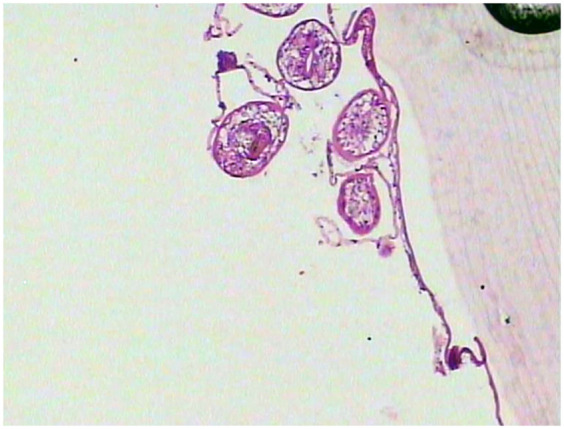
Histological analysis of H&E-stained liver and kidney sections showed hydatid cysts, characterized by a three-layered wall: an inner germinal layer of densely packed cells with round nuclei, a uniform acellular middle laminated layer, and an outer fibrous layer of dense collagen fibers staining dark blue. The cyst cavity contains anechoic fluid.

**Figure 4:**
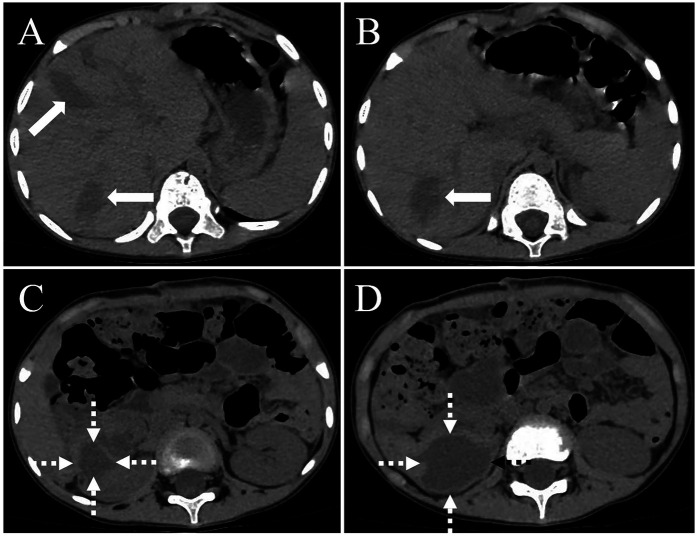
A computed tomography (CT) scan of the abdomen, conducted one month postoperatively, revealed a minor effusion in the surgical region, including within the hepatic parenchyma [**(A,B)**, thick white arrow] and the right renal area [**(C,D)**, white dashed arrow]. There was no indication of echinococcosis recurrence in the surgical site.

On February 10, 2025, a 5-year-old boy from a rural region of Tibet, China, where yak herding constitutes the predominant economic activity, was admitted to the hospital following the identification of a palpable abdominal mass one month prior. The mass, situated in the right lower quadrant of the abdomen, measured approximately 5 cm in diameter and exhibited a slightly firm consistency. The patient exhibited no symptoms of nausea, vomiting, abdominal pain, or other gastrointestinal disturbances. Furthermore, there were no indications of jaundice or superficial lymphadenopathy. His medical history was unremarkable, and there was no reported family history of echinococcosis. Laboratory investigations revealed a normal complete blood count and liver function tests. However, enzyme-linked immunosorbent assay (ELISA) testing revealed the presence of specific IgG antibodies in the serum. Computed tomography (Figure 1) demonstrated multiple hypodense lesions distributed throughout the liver, with the largest lesion in the posterior segment of the right lobe measuring approximately 72 × 55 mm, which did not exhibit enhancement on contrast imaging. The inferior vena cava exhibited compression and narrowing, accompanied by partial occlusion of the right kidney. Based on the imaging and serological assessments, a preliminary diagnosis of complex hepatic echinococcosis and renal cyst was established. A comprehensive multidisciplinary team (MDT) discussion was conducted, involving pediatricians, anesthesiologists, urologists, and infectious disease specialists. The following considerations were made: (1) Infectious Disease Department: Due to the patient's young age, prolonged disease duration, and the substantial size of the parasitic cysts, oral albendazole was deemed to have limited efficacy and posed significant potential side effects. Consequently, surgical intervention was prioritized. (2) Hepatobiliary Surgery: The patient presented with multiple hepatic lesions. To achieve radical treatment, pericystectomy was identified as the preferred surgical approach. Despite the patient's classification as Child-Pugh A in terms of preoperative liver function, hepatic inflow occlusion was avoided during surgery to preserve liver function, given the patient's young age. (3) Urology: The preoperative assessment of the renal cystic lesion presented challenges in accurately determining its nature. The differential diagnoses considered included a simple cyst, hydronephrosis, and renal echinococcosis. In the event that a simple renal cyst was identified intraoperatively, the planned intervention involved cyst puncture and drainage. Conversely, if an echinococcal infection of the right kidney was confirmed, the strategy to preserve renal function prioritized endocystectomy, while nephrectomy was not contemplated. (4) Anesthesiology: The intraoperative anesthesia management strategy focused on maintaining central venous pressure (CVP) above 5 cm H₂O, with the objective of mitigating hepatic ischemia-reperfusion injury. (5) Postoperative Care: The patient was scheduled for transfer to the pediatric intensive care unit to facilitate close monitoring of liver function and to prevent potential complications. Under general anesthesia, the patient underwent a radical multiple pericystectomy of the liver, in conjunction with an endocystectomy of a right renal hydatid cyst, as well as biliary repair and reconstruction (Figure 2). Intraoperative exploration revealed multiple firm, translucent masses located in segments II, III, IV, and VII of the liver, each encapsulated by intact cyst membranes. These masses were found to be closely adherent to the first and second hepatic portal veins, with the inferior vena cava observed to be compressed and narrowed. Additionally, multiple small nodules were noted protruding from the liver surface; however, the diaphragm remained uninvolved. Upon resection and incision of the masses, the contents were identified as powdery, skin-like tissue and brownish-yellow fluid, which communicated with the biliary tract. No nodules were detected in the omentum, abdominal wall, or intestines, and there was an absence of ascites. A large cystic, translucent mass was also identified in the right kidney, which was poorly demarcated from the surrounding renal tissue. Histopathological examination confirmed infection with *Echinococcus granulosus* (Figure 3). In the postoperative period, the patient experienced transient hypernatremia (sodium 165.7 mmol/L) and hepatic dysfunction. Hepatoprotective therapy was initiated, and hypernatremia was effectively managed through the administration of a 5% glucose solution and furosemide. Subsequently, the patient underwent treatment with albendazole (100 mg per dose, administered twice daily) and exhibited a favorable recovery trajectory. At the one-month postoperative follow-up, the patient presented with no signs of jaundice in the skin or sclera. The abdominal examination revealed a soft abdomen without tenderness. Laboratory evaluations, including liver and kidney function tests and complete blood cell counts, were within normal limits. An abdominal CT scan showed no evidence of echinococcosis recurrence (Figure 4).

## 3 Discussion

Simultaneous hepatic and renal echinococcosis in child is exceedingly uncommon, with the majority of case reports concentrating on single-organ involvement (3, 4). This particular case, which involves both the liver and kidneys, not only complicates the diagnostic and therapeutic processes but also underscores the exceptional rarity of such a condition. The presence of lesions in multiple organs may be attributed to the underdeveloped immune system in children, potentially predisposing them to multi-organ involvement (5, 6). Due to the smaller size of pediatric organs, early detection and intervention are imperative to avert complications. Comprehensive collection of medical history is essential for early diagnosis, particularly in patients originating from endemic regions (7). Differential diagnosis from other conditions, such as neoplasms, abscesses, cysts, and tuberculosis, is necessary (8, 9). Imaging modalities, including ultrasound and computed tomography (CT), along with serological testing, are crucial for the accurate diagnosis of echinococcosis (10–13). Surgical intervention is the primary therapeutic approach for echinococcosis. Nonetheless, in child with multi-organ involvement, the surgical risk is elevated, necessitating personalized treatment strategies to ensure complete lesion excision while minimizing complications (14). In the present case, the hepatic lesion was addressed using pericystectomy, a procedure that entails dissection between the inner and outer cyst layers for cyst removal. This technique is less invasive compared to hepatectomy but poses risks such as cyst rupture, dissemination, and biliary fistula formation. Renal echinococcal cysts are relatively uncommon, comprising only 2% of all echinococcal cyst cases. The primary objective in managing renal echinococcosis is to avert renal tissue destruction, allergic reactions, and parasite dissemination. The surgical approach ranges from simple cyst excision to nephrectomy, contingent upon the cyst's size and the extent of tissue damage. In this instance, given the patient's young age and the elevated risk associated with nephrectomy, a nephron-sparing pericystectomy for cystic echinococcosis was conducted. Although this method is straightforward and practical, it is associated with a relatively higher recurrence rate. Postoperative transient hypernatremia in child has been documented in the literature and may, in some instances, result in neurological complications such as epilepsy (15). This highlights the critical need for meticulous postoperative monitoring and timely management of complications to enhance patient outcomes. Given the rarity and complexity of the disease, preventive measures are of paramount importance. The disease predominantly affects pastoral regions and is associated with *Echinococcus granulosus*. Preventive strategies should emphasize public health education, awareness-raising initiatives, minimizing contact with dogs, and improving water hygiene (16). Routine physical examinations and early screening are essential for timely detection and intervention. Future research should prioritize reducing surgical complexity, enhancing precision, evaluating postoperative risks, and optimizing treatment protocols through the use of advanced imaging and navigation technologies (17, 18).

## 4 Conclusions

This case of concurrent hepatic and renal echinococcosis in a child highlights the complexity and rarity of multi-organ involvement in echinococcosis. Early diagnosis through detailed medical history, imaging studies, and serological assays is crucial for timely intervention. The multidisciplinary approach, including tailored surgical strategies and postoperative management, was essential for achieving a favorable outcome. The success of pericystectomy for both hepatic and renal lesions underscores the importance of organ-sparing techniques in child. However, the recurrence risk and potential complications, such as transient hypernatremia, emphasize the need for vigilant postoperative monitoring and supportive care. This study is constrained by specific limitations. The absence of molecular analysis and genotyping on the samples collected post-extraction of the hydatid cysts has hindered the complete utilization of these data to enhance epidemiological research, inform clinical management, and guide public health strategies. Future investigations should incorporate these analyses to achieve a more comprehensive understanding of the characteristics and transmission dynamics of echinococcosis.

## Data Availability

Publicly available datasets were analyzed in this study. This data can be found here: None.
